# Hepatitis B Virus Protein X Induces Degradation of Talin-1

**DOI:** 10.3390/v8100281

**Published:** 2016-10-19

**Authors:** Maarten A. A. van de Klundert, Maartje van den Biggelaar, Neeltje A. Kootstra, Hans L. Zaaijer

**Affiliations:** 1 Department of Blood-borne Infections, Sanquin Research, 1006 AD, Amsterdam, The Netherlands; maarten.van-de-klundert@tum.de (M.A.A.v.d.K.); h.l.zaaijer@amc.uva.nl (H.L.Z.); 2 Department of Experimental Immunology, Landsteiner Laboratory, and Center for Infectious Diseases and Immunity Amsterdam (CINIMA), Academic Medical Center of the University of Amsterdam, 1105 AZ, Amsterdam, The Netherlands; 3 Department of Plasma Proteins, Sanquin Research, 1006 AD, Amsterdam, The Netherlands; m.vandenbiggelaar@sanquin.nl

**Keywords:** hepatitis B virus, protein X, TLN1, HBx, HBV, viral restriction, HBV transcription

## Abstract

In the infected human hepatocyte, expression of the hepatitis B virus (HBV) accessory protein X (HBx) is essential to maintain viral replication in vivo. HBx critically interacts with the host damaged DNA binding protein 1 (DDB1) and the associated ubiquitin ligase machinery, suggesting that HBx functions by inducing the degradation of host proteins. To identify such host proteins, we systematically analyzed the HBx interactome. One HBx interacting protein, talin-1 (TLN1), was proteasomally degraded upon HBx expression. Further analysis showed that TLN1 levels indeed modulate HBV transcriptional activity in an HBx-dependent manner. This indicates that HBx-mediated TLN1 degradation is essential and sufficient to stimulate HBV replication. Our data show that TLN1 can act as a viral restriction factor that suppresses HBV replication, and suggest that the HBx relieves this restriction by inducing TLN1 degradation.

## 1. Introduction

Hepatitis B virus (HBV) is a member of the *Hepadnaviridae* family, and infects hepatocytes in the human liver. After entry, the core particle releases the partially double stranded HBV DNA genome in the nucleus, where it is subsequently repaired by host enzymes to form the HBV covalently closed circular DNA (cccDNA). This circular DNA template of about 3200 base pairs encodes one accessory protein called X (HBx). HBx expression is essential to initiate and maintain HBV replication in vivo [1,2,3,4]. The mechanism by which HBx supports HBV replication is unknown. HBx expression affects various processes such as apoptosis, the cell cycle, and DNA repair [5,6,7,8,9]. In the absence of HBx, viral RNA transcription from the HBV cccDNA is epigenetically silenced [10,11,12]. In most hepatoma-derived cell lines, such as HepG2 cells, HBx expression stimulates HBV transcription but is not essential [11,13,14,15]. HBx invariably transactivates transcription from circular DNA templates, irrespective of the promoter driving transcription [16,17,18,19]. The capacity to transactivate RNA transcription is related to the in vivo function of HBx [20] and can be used to assess HBx functionality [17].

For its function in vivo, HBx requires an interaction with the damaged DNA binding protein 1 (DDB1) [21,22,23,24,25,26,27]. DDB1 can form a complex with Cullin-4A (Cul4A) and an E3 ring ubiquitin ligase that ubiquitinates substrate proteins, which are subsequently degraded by the proteasome. Various viral accessory proteins, such as simian immunodeficiency virus (SIV) accessory protein viral protein X (Vpx) [28], human immunodeficiency virus (HIV) viral protein R (Vpr) [29], and paramyxovirus simian virus 5 V (SV5-V) protein [30], are known to “hijack” this machinery by acting like a DDB1–Cul4A-associated factor (DCAF) in order to specifically degrade a host protein that restricts viral replication, strongly suggesting that HBx functions in a similar manner. Experimental evidence suggests that the interaction between HBx and DDB1, and the concomitant proteasomal degradation of one or more host proteins, is pivotal to HBx function [17,31,32]. Recently, it has been shown that the structural maintenance of chromosome (Smc)5/6 complex is such a host factor [33].

HBx interferes with proteasomal degradation independent of inducing the degradation of a host protein. Although the necessity of the interaction with DDB1 for the transactivation of HBV RNA transcription has been shown to be relevant for HBV replication in hepatoma-derived cell lines, the effect of proteasome inhibitors on HBV replication is unpredictable in such cell lines [32,34]. In HEK 293 cells, however, proteasome inhibition counteracts HBx-mediated transactivation. We show that HBx transactivates transcription from circular DNA by inducing the proteasomal degradation of a host protein. Therefore, we reason that HEK293 cells may also be suitable for the identification of the putative target, which is degraded upon its interaction with HBx and DDB1. We systematically analyzed HBx-interacting proteins in HEK 293 cells in the presence and absence of HBx expression. This revealed a strong reduction in the amount of talin-1 (TLN1) precipitating with HBx in the presence of wild-type HBx. Subsequent analysis showed that in HEK 293 cells TLN1 was indeed degraded by HBx and suppressed transcription from extrachromosomal circular DNA templates. In hepatoma-derived HepG2 cells in which TLN1 was knocked down, HBV replicated efficiently and independent of HBx expression.

## 2. Materials and Methods

### 2.1. Cell Culture and Transfection

HEK 293 and HEK 293T cells were maintained in Dulbecco’s Modified Eagle Medium (DMEM) without 4-(2-hydroxyethyl)-1-piperazineethanesulfonic acid (HEPES) (LONZA, Basel, Switzerland), supplemented with 10% heat inactivated fetal calf serum, penicillin (100 U/mL), and streptomycin (100 μg/mL) (Gibco pen-strep; Gibco Life Technologies, Carlsbad, CA, USA). HepG2 cells were maintained in William’s Medium E without l-Gln (LONZA), supplemented with 10% v/v inactivated fetal calf serum, 2 mM l-Gln (LONZA), penicillin (100 U/mL), streptomycin (100 µg/mL), and 5 uM dexamethasone (Sigma Aldrich St. Louis, MO, USA). HEK 293T and HepG2 cells were maintained in a humidified 10% CO_2_ incubator at 37 °C, and HEK 293 cells were maintained in a humidified 5% CO_2_ incubator at 37 °C. We used HEK 293 cells in the experiments in which the transactivation of HBx was studied to prevent episomal replication of the vectors by the large T antigen present in HEK 293T cells. For subsequent Western blotting experiments, we used HEK 293T cells because of their higher transfection efficiency. Twenty-four hours before transfection, the cells were plated into 6- or 96-well culture plates. The calcium phosphate method was used for transfection. Briefly, plasmid DNA was diluted in 42 mM HEPES pH 7.2 and 2.5 M CaCl_2_ was added to a final concentration of 0.15 M CaCl_2_. The DNA mixture was added to an equal volume of 2× HEPES buffered saline (HBS) (275 mM NaCl, 10 mM KCl, 1.4 mM Na_2_HPO_4_, 42 mM HEPES pH 7.2) and after a 15 min incubation at room temperature the mixture was added to the cells. Cells were incubated overnight in a humidified 3% CO_2_ incubator at 37 °C and subsequently the medium was replaced. Luciferase activity was determined 48 h after transfection.

### 2.2. Expression Vectors and Lentiviral Transduction

The pHSV-HBx vector expressing herpes simplex virus (HSV) epitope-tagged HBx was previously generated and described [35]. This vector was used to generate pHSV-HBx-R96E using targeted mutagenesis (QuikChange II XL site-directed mutagenesis kit, Bio connect (Agilent Technologies, Santa Clara, CA, USA)), according to the manufacturer’s instructions. The primer pairs used were HBx-R96E-F: 5′-CCCAAGGTCTTACATAAGGAGACTCTTGGAGTCCCAGC-3′, and HBx-R96E-R: 5′-GCTGGGAGTCCAAGAGTCTCCTTATGTAAGACCTTGGG-3′. As a vector control, the empty pcDNA3.1A(-) (Invitrogen, Carlsbad, CA, USA) was used. For the construction of plasmids expressing the fusion protein enhanced cyan fluorescent protein (eCFP)-HBx-HSV, HBx was cloned by polymerase chain reaction (PCR) from the pHSV-HBx construct using primers containing an EcoRI restriction site in the forward primer and a KpnI restriction site in the reverse primer. The PCR product was cloned in-frame with eCFP in pcDNA3.1A(-) vectors. The HBV replication assay was previously described in detail [35]. The following reagent was obtained through the National Institutes of Health (NIH) Acquired Immune Deficiency Syndrome (AIDS) Reagent Program, Division of AIDS, National Institute of Allergy and Infectious Diseases (NIAID), NIH: pBlue3′LTR-Luc from Dr. Rienk Jeeninga and Dr. Ben Berkhout (University of Amsterdam, The Netherlands) [36,37]. The PDE-Luc constructs, expressing firefly luciferase (Luc) under control of the human phosphodiesterase 8A (PDE8A) promoters was previously described [38]. To generate the hEF1α-Luc construct, expressing luciferase under control of the human elongation factor α (hEF1α) promoter, the luciferase gene from the LTR-Luc construct was amplified using the primers F-Luc-BamHI: 5′-GATAGGATCCATGGAAGACGCCAAAAAC-3′, and R-Luc-KpnI: 5′-CTATGGTACCTTACACGGCGATCTTTCC-3′. The amplification product was digested with BamHI and KpnI and ligated after the hEF1α promoter in a lentiviral vector digested with the same enzymes [39]. The TG-Luc construct bearing a luciferase reporter under control of the thyroglobulin promoter was a kind gift of Dr. Aho Ilgun (University of Amsterdam, The Netherlands) [40]. The R9 vector, which contains a 1.2-fold overlength HBV DNA genome (subtype adw) in a pGEM7zf+ backbone, was kindly provided by Dr. Thomas Baumert (University of Strasbourg, France) [41,42]. The R9ΔHBx vector was previously described in [35]. The GFP-TLN1 construct (Addgene plasmid 26724) was derived from Addgene and was previously described [43]. The TLN2-expressing construct was a kind gift from Dr. Iwamoto. The primers to generate the luciferase reporters under control of the different HBV promoters were adapted from Du et al. [44] to make them suitable to the HBV adw subtype. Using these primers, the different promoter and enhancer I sequences were amplified by PCR from the R9 vector. The primers used to amplify the HBV promoters and the HBV enhancer I sequence were: Corepromoter-F: 5′-CCCGAGCTCCAAGGTCTTACATAAG-3′; Corepromoter-R: 5′-CCCAAGCTTTGGAGGCTTGAACAGT-3′; S1promoter-F: 5′-CCCGAGCTCTGCCTTACTTTTGGAAG-3′; S1promoter-R: 5′-CCCAAGCTTTTATATAGAATACCAGCC-3′; S2promoter-F: 5′-CCCGAGCTCTTTGCGGGTCACCATA-3′; S2promoter-R: 5′-CCCAAGCTTCCTGACTGCCGATTGGT-3′; Xpromoter-F: 5′-CCCGAGCTCTGCGTGGAACCTTTGT-3′; Xpromoter-R: 5′-CCCAAGCTTGGAAACGATGTATATT-3′; HBVenhancer1-F: 5′-GGGGGTACCGTATACAAGCTAAACA-3′; HBVenhancer1-R: 5′-CCCGAGCTCTGCGCTGATGGCCTATGG-3′. The PCR products containing the HBV promoters were cut with SacI and HindIII and ligated in front of the luciferase reporter of the pGL3Basic vector (Promega, Madison, WI, USA). The constructs that also contained the HBV enhancer I sequence were generated from these constructs by cloning of HBV enhancer I containing PCR product cut with KpnI and SacI in the respective vectors cut with the same enzymes. All constructs were validated by sequencing the BigDye Terminator v1.1 Cycle Sequencing kit (ABI Prism, Applied Biosystems, Foster City, CA, USA), according to the manufacturer’s protocol on a 3730xl DNA analyzer (Applied Biosystems). Sequencing conditions were 5 min at 94 °C, 45 cycles of 15 s at 94 °C, 10 s at 50 °C, 2 min at 60 °C, and a 10 min extension at 60 °C. The short hairpin RNAs (shRNAs) targeting TLN1 (#1: TRCN0000123104, #2: TRCN0000123105 and #3: TRC0000123107) and the control shRNA (SHC001) in a pLKO.1 backbone were derived from the MISSION™ TRC-Hs 1.0 library (Sigma-Aldrich, St. Louis, MO, USA). shRNA #1 and control shRNA were also used to generate the VSV-G-pseudotyped lentiviruses by cotransfection of 1 µg of these constructs together with 0.67 µg pCMV-VSV-G, 0.47 µg pREV, and 1.22 µg pMDL per well in a six-well plate. Twenty-four hours after the medium change, the supernatant was harvested and filtered. HepG2s were transduced in six-well plates for 24 h. Subsequently, the medium was changed and two days later the cells were reseeded for transfection experiments.

### 2.3. Luciferase Assay

Luciferase activity was measured 48 h after transfection by adding 25 μL substrate (0.83 mM ATP, 0.83 mM D-luciferin (Duchefa, Haarlem, The Netherlands), 18.7 mM MgCl_2_, 0.78 μM Na_2_H_2_P_2_O_7_, 38.9 mM Tris pH 7.8, 0.39% (v/v) glycerol, 0.03% (v/v) Triton X-100, and 2.6 μM dithiothreitol) directly to the culture medium. Luminescence was measured for 1 s per well using a luminometer (Berthold, Bad Wildbad, Germany). All experiments were performed in triplicate. The amount of DNA transfected per well was kept constant by cotransfection of our vector control.

### 2.4. Quantitative PCR and Quantification of HBV Replication in HepG2 Cells

To assess the effect of HBx and the different shRNA constructs on PDE8A and TLN1 mRNA, HEK 293T cells were transfected in a six-well plate with 200 ng per well of a vector control or pHSV-HBx. Forty-eight hours after transfection, total RNA was purified using TriPure Isolation Reagent (Roche, Basel, Switzerland) according to the manufacturer’s instructions. cDNA was prepared using random hexameres (Invitrogen) and 200 U M-MLV reverse transcriptase (Promega) in the presence of 20 U RNAsin (Promega) according to the manufacturer’s instructions. Subsequently, 2 µL of cDNA was used in a quantitative PCR using the SYBR Green I Master (Roche) and a LightCycler^®^ 480 system (Roche). PDE8A cDNA was quantified relative to β-actin cDNA using the primers TLN1-F: 5′-CTGGTGCAGAGCTGCAAGGC-3′ and TLN1-R: 5′-CACTGACTCAGCTGCATGGC-3′ for TLN1, PDE8A-1 F: 5′-CGTTTTATACAGTATGCAAATCCT-3′ and PDE8A-1 R: 5′-GCTTTGACGTCTAGTGAGCC-3′ for PDE8A and BA-F: 5′-GGCCCAGTCCTCTCCCAAGTCCAC-3′ and BA-R: 5′-GGTAAGCCCTGGCTGCCTCCACC-3′ for β-actin. The following program was used for quantitative PCR (qPCR): 10 min 95 °C, followed by 50 cycles of 10 s 95 °C, 20 s 59 °C, 30 s 72 °C with a single acquisition during the 72 °C step. The quantification of HBV replication after initiation using the R9 construct was previously described in detail [35]. Briefly, HBV replicating HepG2 cells were lysed in iso-osmotic lysis buffer. After removal of cell nuclei and debris, lysates were treated with DNase to remove non-encapsidated DNA before the number of HBV DNA genome equivalents (geq) was assessed by qPCR.

### 2.5. Western Blotting

For Western blotting, proteins were immunoprecipitated or cells were treated as indicated and subsequently dissolved in radioimmunoprecipitation assay (RIPA) buffer (150 mM NaCl, 1% Triton X-100, 0.5% sodium deoxycholate, 0.1% SDS, 50 mM Tris, pH 8.0) supplemented with Complete^®^ ethylenediaminetetraacetic acid (EDTA) free protease inhibitor (Roche). Immunoprecipitated proteins or treated cell lysates were loaded on a 10% Bis-Tris gel (NuPAGE 10% Bis-Tris precast gel) together with the Odyssey^®^ Protein Weight Marker (LI-COR, Lincoln, NE, USA) and separated by electrophoresis using 2-(*N*-morpholino)ethanesulfonic acid (MES) sodium dodecyl sulfate (SDS) running buffer (Invitrogen). Subsequently, gel-separated proteins were blotted on a nitrocellulose membrane (Protran, Schleicher & Schuell, Dassel, Germany; 2 h, 150V) using NuPAGE transfer buffer. Blots were stained overnight at 4 °C in phosphate-buffered saline (PBS) (Gibco Life Technologies) supplemented with 0.1% Tween-20 (Merck, Kenilworth, NJ, USA) and 5% bovine serum albumin (BSA) (United States Biochemical Corp., Cleveland, OH, USA). The following antibodies were used to visualize proteins: mouse monoclonal anti-DDB1 (1:1000; BD transduction laboratories™); SC-1616 anti-β-actin antibody (1:200; Santa Cruz Biotechnology, Santa Cruz, CA, USA); Mouse monoclonal anti-non-muscle Myosin IIA antibody (1:1000; Abcam, Cambridge, UK, ab55456); mouse monoclonal anti-talin-1 (1:200, Abcam [93E12] ab104913); and rabbit monoclonal anti-talin-2 antibody (1:10,000, Abcam ab108967). IRDye 800CW conjugated goat anti-mouse IgG or 680LT conjugated donkey anti-rabbit (1:15,000; 926–32210, LI-COR) were used as secondary antibodies to visualize expression using the Odyssey^®^ infrared image system (LI-COR). Proteins were quantified using ImageJ (NIH: http://rsbweb.nih.gov/ij/index.html).

### 2.6. Immunoprecipitation and Mass Spectrometry

HEK 293 cells were seeded in six-well plates and transfected with 1 µg of eCFP–HBx expressing vector per well, and cotransfected with 500 ng of empty vector or HSV HBx-expressing vector. Forty-eight hours after transfection, cells from three six-well plates per condition were harvested by trypsin digestion and pelleted at 400 g for 10 min in 50 mL PBS. The cells were lysed in 1 mL of lysis buffer L (20 mM Hepes, 0.5 M NaCl, 1 mM EDTA, 0.25% Triton X-100, 1 mM ethylene glycol-bis(β-aminoethyl ether)-N,N,N',N'-tetraacetic acid (EGTA) supplemented with Complete^®^ EDTA free protease inhibitor (Roche)). Immunoprecipitations were performed using a polyclonal antibody against green fluorescent protein (GFP) (1 µL per IP, AB290, BIO connect) and 25 µL ProtG beads (Thermo Scientific, Waltham, MA, USA). After an overnight incubation at 4 °C rotating, the beads were washed four times with immunoprecipitation (IP) buffer (25 mM Tris, 150 mM NaCl, pH 7.2) supplemented with protease inhibitor (Roche).

Immunoprecipitated proteins were dissolved in 1x sample buffer (NuPAGE LDS loading buffer, Life Technologies, Carlsbad, CA, USA) supplemented with 0.1 M dithiothreitol (DTT), incubated for 5 min at 95 °C, separated on a 4–12% NuPAGE Novex Bis-Tris gel (Life Technologies) and visualized using Coomassie Brilliant Blue (CBB) staining (Imperial Protein Stain, Thermo Scientific). Each gel lane was cut in eight slices and proteins were in-gel digested with trypsin [45]. Peptides were loaded onto Empore-C18 StageTips [46], eluted with 80% acetonitrile (ACN) and 0.5% acetic acid, and analyzed by mass spectrometry (MS).

### 2.7. Mass Spectrometry Data Acquisition

Digested peptides were loaded onto a C18 pre-column (Acclaim Pepmap100, 75 μm × 2 cm, C18 3 μm 100 Å, Thermo Scientific) and separated by a C18 analytical column (Acclaim Pepmap RSLC, 75 μm × 15 cm, C18 2 μm 100 Å, Thermo Scientific) coupled online to a linear trap quadrupole (LTQ)-Orbitrap XL-ETD (Thermo Scientific) via a nanoelectrospray ion source (Nanospray Flex Ion Source, Thermo Scientific) with a spray voltage of 2.0 kV. Peptides were loaded for 10 min at 4% mobile phase (80% ACN, 0.5% acetic acid) and eluted by increasing the mobile phase from 4–30% (10–110 min) and 30–60% (110–135 min), followed by a 5 min wash to 95%, and a 10 min regeneration at 4%. Full scan MS spectra were acquired in the Orbitrap analyzer with a resolution of 60,000 at a mass-to-charge ratio (m/z) of 400, and a target value of 1,000,000 charges. The five most intense precursor ions in the full scan with a charge state of 2+ or higher were selected for collision-induced dissociation (CID) using an isolation width of 2 Da, a 30% normalized collision energy, and an activation time of 30 ms. CID spectra were acquired in the linear ion trap. All data were acquired with Xcalibur software (Thermo Scientific).

### 2.8. Mass Spectrometry Data Analysis

The RAW MS files were analyzed using the MaxQuant computational platform, version 1.3.6.0 [47] and Proteome Discoverer 1.4 software (Thermo Scientific). For Maxquant analysis, proteins and peptides were identified using the Andromeda search engine by querying the human Uniprot database (release-2012 01; 81,213 entries) [48] enabling the ‘match-between-runs’ (MBR) option. To search for precursor and fragment ions, an initial maximal mass deviation of 20 ppm and 0.5 Da, respectively, was required. Trypsin with full enzyme specificity and only peptides with a minimum length of seven amino acids were selected. A maximum of two missed cleavages was allowed. Cys carbamidomethylation was set as fixed modification, while Met oxidation and *N*-acetylation as variable modifications. For protein and peptide identification, we required a maximum false discovery rate (FDR) of 1%. Reverse and contaminant hits were eliminated from the output files. For Proteome Discoverer analysis, peptides were identified using the SEQUEST HT search algorithm by querying the human Uniprot database uniprot-organism-9609-AND-keyword-kw-0181.fasta. In the SEQUEST HT search a precursor mass tolerance of 10 ppm was allowed. Fragment mass tolerance was 0.6 Da. Trypsin with full enzyme specificity and only peptides with a minimum length of seven amino acids were selected. A maximum of two missed cleavages was allowed. Cys carbamidomethylation was set as fixed modification, while Met oxidation and *N*-acetylation as variable modifications. Peptide spectral matches (PSM) were validated using percolator based on q-values at a 1% FDR. With Proteome Discoverer, peptide identifications were grouped into proteins according to the law of parsimony and filtered to 1% FDR.

### 2.9. Software and Statistics

Data from luciferase experiments and qPCR analysis were analysed using GrapPad Prism 5.01 (GraphPad Software, La Jolla, CA, USA). The significance of differences in luciferase activity and mRNA expression was determined by two-sided Student’s *t* testing.

## 3. Results

### 3.1. HBx Transactivates Transcription by Inducing the Degradation of a Host Protein

**Figure 1:**
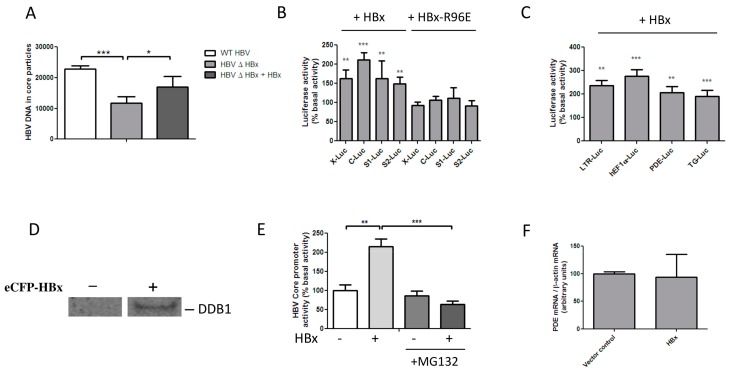
Hepatitis B virus (HBV) accessory protein X (HBx) transactivates transcription by inducing proteasomal degradation of a host protein. (**A**) HBV replication was initiated in HepG2 cells by transfection of a construct containing a 1.2-fold overlength HBV genome that initiates the full HBV replication cycle. The replication of a mutant lacking HBx was impaired but could be rescued by cotransfection of a construct that expresses HBx. (**B**,**C**) Activity of luciferase reporter constructs as a percentage of their basal activities 48 h after transfection in HEK 293 cells. Cells were cotransfected with plasmids expressing HBx or the HBx-R96E mutant, which does not interact with damaged DNA binding protein 1 (DDB1). The luciferase reporters were under control of the hepatitis B core (C), X (X), S1 (S1) and S2 (S2) promoters, or the HIV-1 LTR (LTR), the human elongation factor 1α (hEF1α), the phosphodiesterase 8A (PDE8A), or thyroglobulin (TG) promoters. (**D**) eCFP-HBx was expressed in HEK 293-T cells and lysates were immunoprecipitated with anti-green fluorescent protein (GFP). A Western blot of sodium dodecyl sulfate polyacrylamide gel electrophoresis (SDS-PAGE) separated proteins was stained with anti-DDB1, showing that in these cells HBx interacts with DDB1. (**E**) Activity of the HBV core promoter 48 h after cotransfection with HBx in the absence and presence of proteasome inhibitor MG132, showing that transactivation by HBx critically depends on proteasomal degradation. (**F**) To assess the effect of HBx expression on integrated promoters, we analyzed the PDE8A mRNA production in HEK 293 cells after transfection with HBx. HBx expression did not affect the RNA transcription from the integrated PDE8A promoter, showing that transactivation by HBx is specific for extrachromosomal templates. * *p* < 0.05, ** *p* < 001, *** *p* < 0.001.

HBx expression is essential for HBV replication in vivo, but in HepG2 cells viral replication is possible, although it is attenuated, in the absence of HBx [1,2,3,4,11,13,14,15]. We induced HBV replication in HepG2 cells by transfection of the R9 and the R9ΔHBx vector, which contain 1.2 copies of the HBV genome and initiate HBx proficient and HBx deficient replication, respectively. We assessed the intracellular production of encapsidated HBV DNA to determine HBV replication efficiency [41,49]. Regarding the attenuated replication after transfection of R9ΔHBx, HBV replication could be restored by cotransfection of our pHSV-HBx vector, showing that functional HBx is expressed from this vector [35] (Figure 1A). The promoter-independent transactivation of transfected luciferase reporters can be used to assess HBx functionality [17,20,49]. To investigate under which conditions HBx can transactivate transcription in HEK 293 cells, we transfected HEK 293 cells with luciferase reporters. Cotransfection of an HBx-expressing construct significantly increased the transcriptional activity of luciferase reporters under control of the endogenous HBV promoters (Figure 1B) and several unrelated promoters (Figure 1C). Transactivation by HBx did not depend on the promoters’ basal activity (Figure S1A) or the presence of HBV enhancer 1 (Figure S1B), and maximum transactivation was already reached by the lowest HBx concentration tested (Figure S1C–E). Although HBx interacted with DDB1 in HEK 293 cells (Figure 1D). The well described HBx-R96E mutant, which does not interact with DDB1, failed to transactivate luciferase reporters in HEK 293 cells (Figure 1B and Figure S1B–E). HBx also failed to transactivate promoters in the presence of proteasome inhibitor MG132, confirming that proteasomal degradation is required for transactivation by HBx [25] (Figure 1E). While HBx efficiently transactivated a PDE8A promoter-driven luciferase reporter, we found that PDE8A mRNA levels were not affected by HBx, indicating that transcription from the chromosomal promoter was not affected (Figure 1F). These findings indicate that in HEK 293 cells, as in hepatoma-derived cell lines [17], the transcriptional transactivation by HBx is specific for extrachromosomal templates and involves the proteasomal degradation of a host protein.

### 3.2. HBx Induces the Degradation of Talin-1

**Figure 2:**
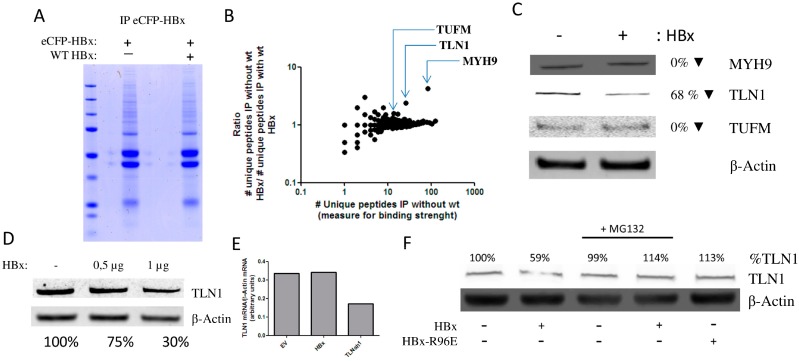
HBx induces proteasomal degradation of talin-1 (TLN1). (**A**) HEK 293 cells were transfected with enhanced cyan fluorescent protein (eCFP)-HBx or eCFP-HBx + wild-type (WT) HBx and lysed 48 h post transfection. eCFP-HBx was immunoprecipitated and interacting proteins were separated by SDS-PAGE and visualized using colloidal blue staining. (**B**) Scatter plots of the ratio of the number of unique peptides in the immunoprecipitation (IP) in the absence/presence of HBx as a function of the number of unique peptides in the immunopreciptitation in the presence of HBx, based on MaxQuant analysis of mass spectrometry data. (**C**) Western blot analysis of lysates of HEK 293T cells stained with antibodies against the indicated proteins. HBx expression resulted in a 68% reduction in TLN1 but did not affect myosin heavy chain 9 (MYH9) or elongation factor Tu, mitochondrial (TUFM) expression. (**D**) Western blot analysis of TLN1 level after transfection with increasing amounts of HBx; (**E**) HEK 293T cells were transfected with an empty vector (EV), or with constructs expressing either HBx or a short hairpin RNA (shRNA) against TLN1. While HBx expression did not affect TLN1 mRNA production, expression of the short hairpin RNA (shRNA) against TLN1 (TLNsh1) resulted in mRNA degradation. (**F)** Western blot analysis of lysates of HEK 293T cells stained with antibodies against the indicated proteins. HBx-mediated degradation could be prevented by adding proteasome inhibitor MG132. The HBX-R96E mutant did not affect TLN1 levels. In all Western blot experiments, protein levels are given as quantified by Image J as a percentage of the levels in the absence of HBx in the same experiment, relative to β-actin.

Previously, the Smc5/6 complex was identified as a host factor targeted by HBx by means of coprecipitation of HBx-interacting proteins with a fusion construct of HBx and DDB1 proteins that were pro- and deficient in interacting with Cul4A [33]. To identify other HBx-interacting proteins for further analysis, we generated a eCFP-HBx fusion protein. While eCFP-HBx was efficiently expressed (Figure S2A), it did not transactivate transcription from extrachromosomal templates in HEK 293 cells (data not shown). When eCFP-HBx was precipitated using an anti-GFP antibody that interacts with eCFP, we observed that the eCFP-HBx fusion protein could still coprecipitate the HBx interacting protein DDB1 (Figure 1D). These results indicate that the eCFP-HBx fusion protein may still interact with the protein targeted for degradation, but that steric hindrance of the eCFP protein would prevent the formation of the HBx–DDB1–Cul4 complex required to induce its proteasomal degradation. To further test this hypothesis, we analysed all proteins precipitating with eCFP-HBx. To identify the putative target of HBx, interacting proteins were precipitated with eCFP-HBx in the presence and absence of wild-type HBx, reasoning that this protein should be enriched in cells not coexpressing wild-type HBx. eCFP-HBx coprecipitating proteins were separated by sodium dodecyl sulfate polyacrylamide gel electrophoresis (SDS-PAGE) and visualized by colloidal blue staining (Figure 2A). We analysed all proteins that precipitated with eCFP-HBx using mass spectrometry. Peptides from 2376 (MaxQuant analysis, Table S1) and 2885 (Proteome Discoverer analysis; see Table S2) proteins were identified. Of the identified proteins, heat shock 70 kDa protein 1A/1B (HSP1A/HSP1B), which was previously described to interact with HBx [50], was the protein that most abundantly coprecipitated with eCFP-HBx in both conditions (Figure S2B). For several proteins the number of identified peptides was enriched in the immunoprecipitation in the absence of wild-type HBx (Figure 2B and Figures S2B,C). This suggested that these proteins are possible targets for HBx–DDB1-mediated degradation. The three proteins that were most enriched in the absence of wild-type HBx and for which more than 10 unique peptides were detected, were selected for further analysis: myosin heavy chain 9 (MYH9), TLN1, and elongation factor Tu, mitochondrial (TUFM). We observed that wild-type HBx expression in HEK 293T cells did not induce the degradation of MYH9 or TUFM. This suggests that wild-type HBx may compete with eCFP-HBx for binding to MYH9 and TUFM, but that wild-type HBx did not induce their proteasomal degradation (Figure 2C). When we expressed HBx in HEK 293T cells, we observed a reduction in TLN1 protein levels (Figure 2C). TLN1 was previously found to interact with HBx [51]. TLN1 degradation by HBx was confirmed in additional experiments, though TLN1 degradation required relatively high levels of HBx expression (Figure 2D). TLN1 mRNA expression levels were not affected by HBx expression, showing that the decrease in TLN1 level results from increased proteasomal TLN1 degradation (Figure 2E). Expression of the HBx-R96E mutant did not affect TLN1 levels, and the addition of proteasome inhibitor MG132 prevented HBx-mediated TLN1 degradation (Figure 2F). This confirms that HBx needs to interact with DDB1 to induce proteasomal degradation of TLN1. In addition, the levels of TLN2, which is highly homologous to TLN1 (74% amino acid sequence identity), were not affected by HBx expression (Figure S3A).

### 3.3. Talin-1 Suppresses HBV Replication by Interfering with RNA Transcription

**Figure 3:**
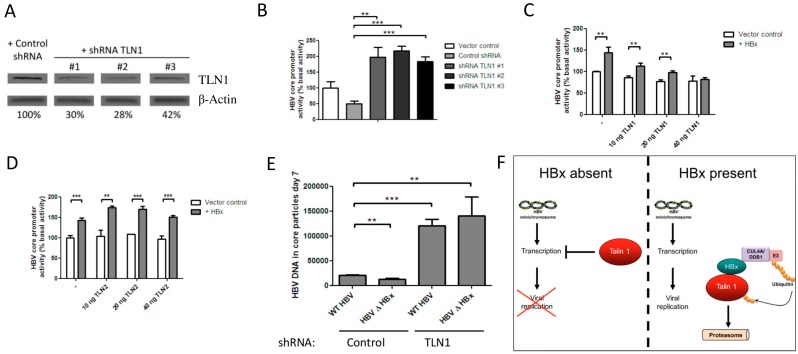
Talin-1 interferes with HBV replication by suppressing RNA transcription. (**A**) Western blot of lysates of HEK 293-T cells 48 h after transfection with plasmids expressing shRNAs against TLN1, showing that all shRNAs against TLN1 reduce the TLN1 protein level. (**B**) HEK 293 cells were transfected with a luciferase reporter under control of the HBV core promoter, and cotransfected with the plasmids expressing shRNAs against TLN1, showing that TLN1 knockdown efficiently transactivates transcription. (**C**) Cotransfection of a vector expressing a biologically active GFP-TLN1 fusion protein with a luciferase reporter under control of the HBV core promoter prevented transactivation by HBx in a dose-dependent manner. (**D**) Activity of a luciferase reporter under control of the HBV core promoter 48 h after cotransfection with a construct expressing TLN2. TLN2 overexpression did not affect transactivation by HBx. (**E**) HBV DNA in core particles in the cytoplasm of the cells was analyzed seven days post-transfection (HBV genome equivalents (geq)/μL). HepG2 cells were transduced with a lentivirus expressing the shRNA against TLN1. HBV replication was initiated by transfection of the 1.2-fold overlength HBV containing R9 vector or its mutant (R9ΔHBx) lacking HBx expression. (**F**) A schematic representation of HBx function. ** *p* < 0.01, *** *p* < 0.001.

Next, we analyzed whether TLN1 is indeed a host protein restricting the transcription from our luciferase reporters in HEK 293 cells. To reduce TLN1 protein levels, HEK 293 cells were transfected with plasmids expressing shRNAs targeting TLN1. shRNA expression induced a strong reduction of TLN1 mRNA (Figure 2E) and protein levels (Figure 3A) 48 h post-transfection. Like HBx expression, shRNA-mediated reduction of TLN1 levels markedly increased transcription from luciferase reporters under control of the HBV core- (Figure 3B), HBV X-, and human PDE8A promoters (Figure S3B,C). Conversely, overexpression of a GFP-TLN1 fusion construct prevented HBx-mediated transactivation of luciferase reporters under control of the HBV core (Figure 3C), and human PDE8A promoters (Figure S3D) in a dose-dependent manner. This effect was highly specific for HBx expression, as GFP-TLN1 overexpression did not affect transcription from these reporters in the absence of HBx. In contrast, overexpression of TLN2 did not affect HBx-mediated transactivation of a luciferase reporter under control of the HBV core promoter (Figure 3D). To assess the effect of TLN1 on the full HBV replication cycle, we generated a lentiviral vector to express TLN1 shRNA #1. HepG2 cells were transduced with this lentiviral vector, resulting in a 55% decrease in TLN1 mRNA (Figure S3E). Upon induction of HBV replication by transfection with the R9 or the R9ΔHBx vector, the TLN1 knockdown cells produced five-fold more HBV DNA in core particles than HepG2 cells transfected with the R9ΔHBx vector (Figure 3E). Replication of HBV in the presence of functional HBx also increased in the TLN1 knockdown cell line. This may be due to the in vitro conditions, which have been reported to distort TLN1 mediated processes and the related signalling pathway. In TLN1 knockdown cells, HBx was unable to augment HBV replication, suggesting that the HBx mediated TLN1 degradation is sufficient to augment HBV replication in HepG2 cells.

## 4. Discussion

Expression of HBx is required for in vivo HBV replication. HBx interacts with DDB1, a host protein often “hijacked” by viral accessory proteins to induce the degradation of a host protein that interferes with viral replication [22,23,24,25,26,27,28,29,30,31,33]. We observed that in HEK 293 cells, HBx-mediated transcriptional transactivation required interaction with DDB1 and proteasomal degradation. We identified HBx-interacting proteins using an eCFP-HBx fusion protein, which was able to interact with DDB1 but did not transactivate transcription and therefore presumably prevents the formation of the DDB1-associated complex that would otherwise induce the degradation of the putative target of the HBx–DB1 interaction. By applying subtractive mass spectrometry, we identified TLN1 as an HBx-interacting protein that is degraded in the presence of HBx in these cells. Recently, the Smc5/6 complex was also identified as an HBx-interacting host factor that is degraded upon interaction with HBx. In these experiments HBx was linked to DDB1, which may induce conformational changes necessary for the interaction with Smc5/6, as this complex was not identified in our experiment. Although our method may not enable precipitation of all HBx-interacting proteins, and we cannot be certain that the fusion to eCFP prevents the formation of all complexes containing target proteins, HBx and DDB1, proteins that do precipitate with eCFP-HBx, are HBx-interacting proteins. Furthermore, the interaction between HBx and TLN1 has been previously observed by others [51]. Also, HBx expression may interfere with the interaction of eCFP-HBx with other HBx-interacting proteins not targeted for proteasomal degradation, which may be the case for MYH9 and TUFM. Disruption of TLN1 levels by shRNA-mediated knockdown or by HBx expression enhanced transcription driven by viral and non-viral promoters. HBx-transactivated transcription could be restored to basal levels by overexpression of GFP-TLN1. In the absence of HBx, transcription was not affected by similar levels of TLN1 overexpression. When we initiated the full HBV replication cycle in HepG2 cells, TLN1 knockdown stimulated HBV replication and prevented HBx from further augmenting viral replication. TLN1 knockdown stimulated HBV replication more efficiently than HBx expression, which may be due to the in vitro extracellular environment, which heavily distorts the dynamics of TLN1-mediated adhesion processes. However, we cannot exclude that TLN1 knockdown affects other aspects of the viral life cycle that are not targeted by HBx. These data suggest that HBx-mediated TLN1 degradation relieves an inhibition on HBV RNA transcription, as schematically represented in Figure 3F. Initiation of HBV replication in HepG2 cells by transfection results in cccDNA formation and HBx-dependent replication [52], but circumvents the natural infection steps (viral entry), and may not recapitulate HBx-mediated effects early in the HBV replication cycle that depend on natural infection [15]. Thus, to establish TLN1 as a viral restriction factor counteracted by HBx, it would be necessary to confirm TLN1 degradation, and demonstrate the effects of TLN1 levels on HBV replication in primary hepatocytes naturally infected with HBV.

TLN1 links the cytoplasmic domain of β integrins to the actin cytoskeleton and is involved in the formation of focal adhesions [53,54]. The dynamics of these structures, and the activities of associated pathways, are heavily influenced by ex vivo conditions, which may explain why in HepG2 cells TLN1 knockdown stimulated HBV replication more efficiently than HBx expression. Like HBx, TLN1 has been involved in a broad range of processes such as cell viability and apoptosis, proliferation, migration, signal transduction, and transcription. In the cytoplasm, TLN1 forms a compact, auto-inhibiting heterodimer in which the active domains are shielded [55]. TLN1 is ubiquitinated and proteasomally degraded during dynamic cell processes [56]. Upon activation and monomerisation, TLN1 links the cytoplasmic domain of β integrins to the actin cytoskeleton, a fundamental process in the formation of focal adhesions [53,54,57]. In vitro, the dynamics of these complexes, and hence the TLN1 biology, are disturbed and poorly mimic the in vitro behavior. Notably, it has been observed that HBx expression disrupts the formation of focal adhesions [58,59] and modulates the activity of the associated focal adhesion kinase [60].

It is unclear how TLN1 suppresses HBV RNA transcription, or how HBx-mediated TLN1 degradation would modulate cellular behavior. The changes in viral and cellular gene transcription may result from the altered dynamics of focal adhesions and the related signalling pathways. Several findings suggest that TLN1 can indeed affect transcription by modulating interactions and signalling processes in focal adhesions at the cell membrane, which seem fundamentally involved in transcriptional regulation [61,62]. In *Drosophila*, TLN1 strongly suppresses the DE-cadherin and shotgun promoters [63,64]. Notably, it has been shown that various cytoplasmatic signalling pathways are deregulated by HBx, and it seems unlikely that such distortions would result from the degradation of host factors that directly interact with the cccDNA. The involvement of TLN1 in HBV replication suggests that the lack of interaction between integrins and a realistic extracellular matrix in vitro may contribute to the large difference between the effects of HBx expression on HBV replication in vitro and in vivo [13]. The lack of such interaction and the concomitant lack of TLN1 dynamic behavior may also explain why TLN1 knockdown stimulates HBV replication more efficiently in HepG2 cells than HBx-mediated degradation. Integrin signalling may indeed affect HBV replication in vivo, as it has been observed that genetic polymorphisms in the integrin αv gene are associated with HBV chronicity and hepatocellular carcinoma (HCC) [65].

It has been described that HBx is recruited to the cccDNA by host proteins, and that HBx enhances the transcriptional activity locally by modulating the recruitment of epigenetic regulatory proteins [10,11,12,66]. During cytokinesis, TLN1 associates with the chromosome centromeres [67,68], indicating that TLN1 is principally capable of interacting with defined protein–DNA complexes in the nucleus. This could indicate that TLN1 or fragments thereof may directly interact with the cccDNA. Thus, to establish how TLN1 suppresses HBV RNA transcription, it would be vital to establish whether TLN1 directly associates with the HBV cccDNA and recruits transcriptional repressors to it [10,66].

Chronic infection with HBV is the major aetiological agent associated with hepatocellular carcinoma (HCC), and various lines of evidence suggest that HBx is involved in oncogenic transformation. In HCC, HBx is frequently expressed from chromosomally integrated HBV DNA fragments [69], and several transgenic mouse models that express HBx in their livers spontaneously develop HCC [70,71]. *TLN1* is frequently overexpressed in various types of cancer, and serum TLN1 levels have been associated with HCC progression. Interestingly, the amount of TLN1 in HCC tissue varies. TLN1 expression correlates with HCC dedifferentiation [72]. Notably, HBV replication is inversely correlated to the hepatocyte differentiation state [73].

TLN1 has previously been implicated to play a role in the replication of other viruses. TLN1 suppresses the replication of retroviruses, although the inhibition is not related to viral transcription [74]. The human cytomegalovirus protein pUL135 was shown to interact with TLN1 and to disrupt the interaction between infected cells and the extracellular matrix [75]. In leukocytes, TLN1 is critically involved in the initiation of adaptive immune responses by activating integrins following cytokine- [76] or T cell-receptor stimulation [77,78].

Here, we identified TLN1 as a viral restriction factor that suppresses HBV replication by interfering with viral RNA transcription. HBx relieves this restriction by inducing TLN1 degradation. Knowledge of this mechanism may aid the development of antivirals that interfere with HBx and suppress HBV RNA transcription. Such antivirals may complement existing treatments by suppressing the ongoing viral antigen production in chronically infected patients.

## Supplementary Materials

The following are available online at www.mdpi.com/1999-4915/8/10/281/s1, Figure S1: Transcriptional transactivation by hepatitis B virus (HBV) accessory protein X (HBx), Figure S2: Immunoblot control and peptide spectral matches of HBx-interacting proteins, Figure S3: Talin-1 (TLN1) suppresses transcription and is specifically degraded in the presence of HBx, Table S1: Protein groups identified in the immunoprecipitation experiments based on mass spectrometry data analysed by MaxQuant, Table S2: Protein groups identified in the immunoprecipitation experiments based on mass spectrometry data analysed by Proteome Discoverer.
